# Meiotic chromosome dynamics and double strand break formation in reptiles

**DOI:** 10.3389/fcell.2022.1009776

**Published:** 2022-10-12

**Authors:** Laia Marín-Gual, Laura González-Rodelas, Maria M. Garcias, Lukáš Kratochvíl, Nicole Valenzuela, Arthur Georges, Paul D. Waters, Aurora Ruiz-Herrera

**Affiliations:** ^1^ Departament de Biologia Cel·lular, Fisiologia i Immunologia, Universitat Autònoma de Barcelona, Cerdanyola del Vallès, Spain; ^2^ Genome Integrity and Instability Group, Institut de Biotecnologia i Biomedicina, Universitat Autònoma de Barcelona, Cerdanyola del Vallès, Spain; ^3^ Department of Ecology, Faculty of Science, Charles University, Prague, Czech Republic; ^4^ Department of Ecology, Evolution, and Organismal Biology, Iowa State University, Ames, IA, United States; ^5^ Institute for Applied Ecology, University of Canberra, Canberra, ACT, Australia; ^6^ School of Biotechnology and Biomolecular Sciences, Faculty of Science, UNSW, Sydney, NSW, Australia

**Keywords:** reptile, meiosis, gametogenesis, micro-chromosomes, DSBs, recombination, *bouquet*

## Abstract

During meiotic prophase I, tightly regulated processes take place, from pairing and synapsis of homologous chromosomes to recombination, which are essential for the generation of genetically variable haploid gametes. These processes have canonical meiotic features conserved across different phylogenetic groups. However, the dynamics of meiotic prophase I in non-mammalian vertebrates are poorly known. Here, we compare four species from Sauropsida to understand the regulation of meiotic prophase I in reptiles: the Australian central bearded dragon (*Pogona vitticeps*), two geckos (*Paroedura picta* and *Coleonyx variegatus*) and the painted turtle (*Chrysemys picta*). We first performed a histological characterization of the spermatogenesis process in both the bearded dragon and the painted turtle. We then analyzed prophase I dynamics, including chromosome pairing, synapsis and the formation of double strand breaks (DSBs). We show that meiosis progression is highly conserved in reptiles with telomeres clustering forming the *bouquet*, which we propose promotes homologous pairing and synapsis, along with facilitating the early pairing of micro-chromosomes during prophase I (i.e., early zygotene). Moreover, we detected low levels of meiotic DSB formation in all taxa. Our results provide new insights into reptile meiosis.

## Introduction

Meiosis is used by all sexually reproducing organisms to form haploid gametes (oocytes or sperm) *via* two consecutive cell divisions preceded by one round of genome replication. This follows a tightly regulated progression of chromosome condensation and folding, coupled with changes to the epigenome and gene expression (Hammoud et al., 2014; Alavattam et al., 2019; Patel et al., 2019; Vara et al., 2019; Vara and Ruiz-Herrera, 2022). Meiosis generates genetically variable gametes by recombination of the two parental chromosomes during prophase I. This involves faithful chromosome synapsis, the formation of double strand breaks (DSBs) and DNA exchange (crossovers, COs) between homologues.

Meiotic prophase I is commonly subdivided into four different stages (leptotene, zygotene, pachytene and diplotene) based on the dynamics of meiotic chromosomes and their telomeres (reviewed in Bolcun-Filas and Handel, 2018). The pairing of homologous chromosomes begins at leptotene with the formation of a protein scaffold along chromosomes composed of cohesins and proteins specific to the synaptonemal complex (SC). This coincides with the generation of DSBs by the endonuclease protein SPO11 (Keeney et al., 1997). Telomeres play an important role during the leptotene-zygotene transition, clustering to form a structure known as the *bouquet* (Scherthan et al., 1996; Liebe et al., 2004; Reig-Viader et al., 2013). At zygotene, DSBs are repaired, leading to their resolution as either COs or non-COs (NCOs) between sister chromatids. It is not until pachytene that chromosomes are completely synapsed and COs are resolved as chiasmata (the points where genetic material is actually exchanged). The mechanisms underlying meiotic progression have been extensively studied in several model organisms, including yeast, fruit flies, nematodes, mice and zebrafish (Zickler and Kleckner, 2015; Blokhina et al., 2019; Imai et al., 2021). However, our understanding of the dynamics of meiotic prophase I and recombination among non-mammalian amniote vertebrates (i.e., sauropsids–birds/reptiles) remains incomplete (Segura et al., 2013; Marín-Gual et al., 2022).

**FIGURE 1:**
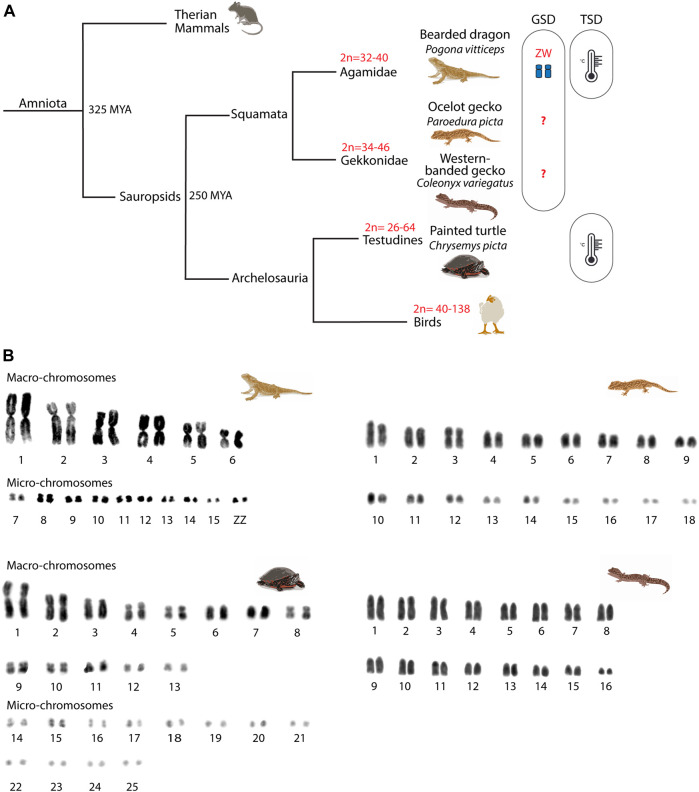
Phylogeny of the reptiles included in the study. **(A)** Phylogenetic relationships of the four reptilian species included in the study. For each phylogenetic branch, variation in diploid numbers and sex determination system are indicated. **(B)** Mitotic karyotypes of the four studied species: *Pogona vitticeps*, *Paroedura picta*, *Chrysemys picta* and *Coleonyx variegatus*. The bearded dragon (*P. vitticeps*) and the painted turtle (*C. picta*) karyotypes include macro- and micro-chromosomes, whereas the western banded gecko (*C. variegatus*) and the ocelot gecko (*P. picta*) karyotypes include chromosomes of progressively smaller size. Karyotypes correspond to mitotic metaphases from fibroblast primary cell cultures. GSD: genotypic sex determination; TSD: temperature sex determination.

Amniote vertebrates shared a last common ancestor approximately 325 mya (Shedlock and Edwards, 2009) (Figure 1) and are characterized by distinctive chromosome morphology and evolutionary labile sex determination. Sauropsids display variation in chromosome number, especially in birds (2n = 40–138), although this is less pronounced in reptiles (2n = 22–68) (Ruiz-Herrera et al., 2012; Montiel et al., 2016; Waters et al., 2021). The non-avian sauropsids (reptiles) are composed of Squamata (lizards and snakes), Sphenodontia (tuatara), Crocodilia (crocodiles and alligators), and Testudines (turtles). Reptiles are characterized by the presence of generally well conserved micro- and macro-chromosomes (Waters et al., 2021) and by a high variability in their sex-determining systems (i.e., ZZ/ZW, XX/XY or temperature sex determination - TSD) (Ezaz et al., 2006) (Figure 1). While meiotic progression in the chicken has been studied and mirrors eutherians (Schoenmakers et al., 2009; Guioli et al., 2012), little is known about meiosis in reptiles. The few existing reports focused on CO formation (Lisachov et al., 2017, 2019; Spangenberg et al., 2021) and formation of unreduced eggs in parthenogenetic lineages (Lutes et al., 2010), but whether meiotic progression in reptiles resembles the process described for either mammals or zebrafish (which last shared a common ancestor with amniotes approximately 400 mya) is currently unknown.

Here we provide a comparative analysis of key features of spermatogenesis and meiotic prophase I progression in previously uncharacterised reptile linages, with a focus on meiotic recombination. We examined the ocelot gecko (*Paroedura picta*) and the western-banded gecko (*Coleonyx variegatus*) as representatives of Gekkota (geckos), the Australian central bearded dragon (*Pogona vitticeps*) as a representative of Iguania (iguanas, agamids and chameleons), and the painted turtle (*Chrysemys picta*) as a representative of Testudines (turtles). These species are emerging models for thermal and reproductive physiology (Valenzuela, 2009; Starostová et al., 2013; Kubička et al., 2015), as well as developmental biology (Noro et al., 2009). The three lizards have genotypic sex determination (GSD). The bearded dragon has ZW sex chromosomes (Ezaz et al., 2005; Koubová et al., 2014), whereas the western banded gecko and the ocelot gecko GSD systems are still unknown (Rovatsos et al., 2019; Keating et al., 2022). However, the genetic sex determination of the bearded dragon can be overridden by temperature to produce viable ZZ females (Quinn et al., 2007; Holleley et al., 2015). Most turtle species have temperature-dependent sex determination (TSD, including *C. picta*), although XY and ZW systems are also present in different lineages (Bista and Valenzuela, 2020) (Figure 1).

Our study unveils shared features between bearded dragon and painted turtle spermatogenesis. We also observed that all reptiles examined here present an equivalent pattern of prophase I progression forming the *bouquet* at early stages, where homologous micro-chromosomes synapse first and cluster together. Remarkably we detected low rates of DSB formation in reptiles when compared to mammals, suggesting that low recombination rates are a distinctive feature of reptiles.

## Material and methods

### Samples

Male bearded dragons (*n* = 4, *P. vitticeps*) were obtained from captive colonies in Canberra (ACT, Australia) at the end of the breeding season (February). Male ocelot geckos (*n* = 3, *P. picta*) and male western banded geckos (*n* = 1, *C. variegatus*) were originated from breeding colonies in Charles University in Prague (Czech Republic). Male painted turtles (*n* = 3, *C. picta*) were wild-caught in Iowa (United States) at the end of the breeding season under appropriate permits from Iowa’s DNR.

### Primary fibroblast cell culture and karyotyping

Four primary fibroblast cell lines were derived from all reptile species studied. Samples of connective tissue were washed in 1xPBS supplemented with an antibiotic-antimycotic solution (100 U/ml penicillin, 100 μg/ml streptomycin, 50 μg/ml gentamicin and 0.25 μg/ml amphotericin B). Cultures were established by disaggregating tissue with a scalpel blade and resuspending cells in AmnioMAX. Cell cultures were incubated at 28°C in 5% CO_2_.

For karyotyping, cells were arrested in metaphase by adding 80 μl of Colcemid (10 μg/ml) to 10 ml of medium for 2 h and then trypsinised. Cells were centrifuged down at 600 xg for 5 min and resuspended in 5 ml of hypotonic solution (0.075M KCl) for 30 min at 37°C. Chromosomes were then fixed by addition of fixative solution (3:1 methanol/acetic acid) and metaphase spreads were obtained by dropping 15 µl of cell suspension onto a cleaned dry slide. Slides were baked at 65°C for one hour and kept at −20°C until use. Metaphases were stained homogenously with DAPI for the karyotype analysis.

### Histology and testis morphometry

Testes from the bearded dragon and the painted turtle were collected for histological procedures. Briefly, testes were fixed overnight in Bouin’s solution (70% saturated picric acid, 25% formaldehyde and 5% glacial acetic acid). Then, samples were dehydrated, cleared and embedded in paraffin using standard procedures. Sections (7 µm) were stained with PAS-hematoxylin.

### Spermatocyte spreads and immunofluorescence

Testicular biopsies were obtained immediately after animal dissection and processed as previously described (Garcia-Cruz et al., 2011) in order to obtain spermatocyte spreads. Briefly, a piece of the testicular biopsy was carefully minced on a slide; 1% Lipsol was added and incubated for 30 min at room temperature. Then, a fixative solution containing 4% paraformaldehyde was added, and slides were kept in a humid chamber. After two hours, slides were washed in 1% photo-flo solution and further processed for immunofluorescence, or frozen at −20°C until use.

Immuno-staining of meiocytes was performed using the following primary antibodies: rabbit antibody against SYCP3 (#ab15093, Abcam, 1:100 dilution), rabbit antibody against SYCP1 (#ab15087, Abcam, 1:100 dilution), rabbit antibody against TRF2 (#NB110-57130SS, Novus Biologicals, 1:100 dilution), mouse antibody against RNA pol II (#5408, Abcam, 1:400 dilution), rabbit antibody against RAD51 (#PC130, Calbiochem, 1:50 dilution), rabbit antibody against RPA32/RPA2 (#10359, Abcam, 1:100 dilution), mouse antibody against MLH1 (#51–1327GR, BD PharmigenTM, 1:100 dilution), rabbit antibody against MLH1 (#ab47703, Abcam, 1:100 dilution) and rabbit antibody against γH2AX (#H5912, Sigma-Aldrich, 1:100 dilution).

Fluorochrome-conjugated secondary antibodies were used for detection (all from Jackson ImmunoResearch Laboratories). Antibodies were diluted in PBST (Tween 0.05% in PBS). Primary antibodies were incubated overnight at 4°C in a humid chamber and secondary antibodies for 1 h at 37°C in a humid chamber. After washing away the excess of secondary antibodies, DNA was counterstained with anti-fade solution (Vectashield) containing 8 μg/ml DAPI (4′,6′-diamidino-2-phenylindole).

### Microscopy and image analysis

PAS-hematoxylin–stained tissue sections were analyzed on an Olympus CH2 microscope, and images were captured using a Zeiss Axiophot Microscope and Olympus C5060 camera. For fluorescent sample analysis and image capturing, a Zeiss Axioskop fluorescence microscope connected to a ProgRes Jenoptik camera was used. The image capture software ProgRes CapturePro was employed for image acquisition and image processing.

The accumulation of foci in the *bouquet* was analyzed as the percentage of foci per cell located in the *bouquet* region, previously delimited as the area where synaptonemal complex (i.e., SYCP3 signal) begins to assemble and SYCP3 intensity is higher. Only cells with a well-defined *bouquet* were included in the analysis.

### Statistical analysis

Statistical significance for the DSB analysis as RPA and RAD51 foci, and for the analysis of the percentage of DSB foci in the *bouquet* was determined using two-sided Mann-Whitney U-tests. The critical value for statistical significance was *p* < 0.05 for all tests. Each plot or its figure legend indicates the statistical methods and corresponding *p*-values. All boxplots are represented as centre lines (median), box limits (interquartile range; 25th and 75th percentiles) and whiskers (largest and lowest data points inside the first and third quartiles plus 1.5 times the interquartile range).

## Results

### Spermatogenesis progression in the bearded dragon and the painted turtle

**FIGURE 2:**
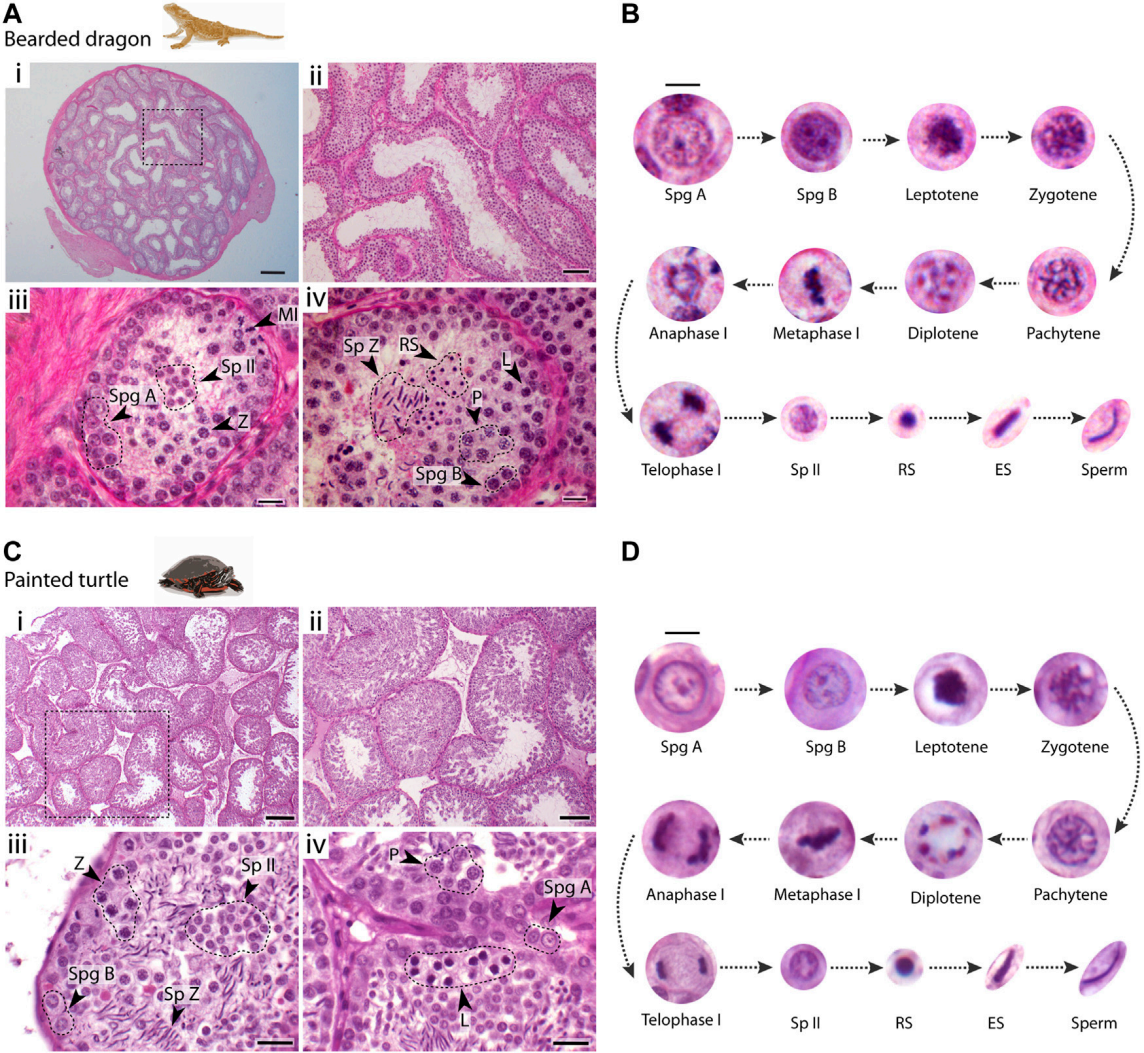
Testis histology. Histological cross-sections of seminiferous tubules of **(A)** the bearded dragon and **(C)** the painted turtle stained with PAS-hematoxylin. Dashed circles and arrowheads point clusters of different cell types. Scale bars: (i) 200 μm, (ii) 100 μm, and (iii, iv) 20 μm. Germ cell types found within the seminiferous epithelium and their progression in **(B)** the bearded dragon and **(D)** the painted turtle. Scale bar: 10 μm. Legend type: Spg A, type A spermatogonia; Spg B, type B spermatogonia; L, leptotene spermatocyte; Z, zygotene spermatocyte; P, pachytene spermatocyte; D, diplotene spermatocyte; MI, metaphase I; Sp II, secondary spermatocyte; RS, round spermatid; ES, elongating spermatid; Sp Z, spermatozoa.

We first characterized spermatogenesis progression in the bearded dragon (*P. vitticeps*) and the painted turtle (*C. picta*) (Figure 2), following the mammalian classification of germ cell morphology (Russell et al., 1993). Both the bearded dragon (Figures 2A,B) and the painted turtle (Figures 2C,D) had a histological organisation of germ cells within the seminiferous epithelia (between the basal lamina and the lumen) that was similar to that of eutherian mammals (Russell et al., 1993) and other amniotes (Gribbins, 2011).

In both species, spermatogonia (A and B) were restricted to the basal lamina (Figures 2A,C). Type A spermatogonia presented a rounded nucleus showing one nucleolus, whereas type B spermatogonia contained densely stained chromatin and more than two nucleoli (between 2-4 nucleoli) (Figures 2B,D). Large populations of cells subsequently progress through meiosis towards the centre of the seminiferous tubule. Meiotic cells were characterized by an increase in size and condensed chromatin. This included recognizable stages of prophase I: leptotene, zygotene, pachytene and diplotene (Figure 2). Both first and second meiotic divisions and secondary spermatocyte stages occurred rapidly, as all three phases were found in low proportions in cross-sections of seminiferous tubules (Figures 2A,C). Leptotene spermatocytes were distinguished by dense filamentous chromatin at the nuclei. Zygotene spermatocytes exhibited clumps of condensed filamentous chromatin within the nucleus. Pachytene spermatocytes displayed an open nucleoplasm and their nuclei contained thick chromatin fibres. Finally, diplotene spermatocytes had chromatin fibres in a tight circle and degenerating nuclear membranes. We also distinguished meiotic cells with the chromosomes fully condensed and aligned at the metaphase plate. During the second meiotic division, secondary spermatocytes contained randomly dispersed chromatin fibres (Figures 2B,D).

Spermiogenesis (the differentiation and maturation of sperm) encompasses a longer period than previous stages as large populations of round and elongating spermatids were observed (Figure 2). Spermiogenic cells were divided into three different stages: i) round spermatids, the smallest cell type, rounded with fully condensed chromatin; ii) elongating spermatids with their round nuclei and condensed chromatin becoming elongated; iii) mature sperm after the completion of spermiogenesis and the elongation process was finalised.

### Micro-chromosomes pair earlier during prophase I than macro-chromosomes

We then analyzed the meiotic chromosome pairing strategies in all four species. Chromosome pairs in all four reptile species largely differ in size (Figure 1). The chromosome complement of the reptiles herein varied: 2n = 32 chromosomes in the bearded dragon (6 pairs of macro chromosomes and 10 pairs of micros, including the sex chromosomes) (Young et al., 2013), 2n = 32 in the western-banded gecko (16 pairs of acrocentric chromosomes with continuous decreasing of size from large to small) (Pokorná et al., 2010), 2n = 36 chromosomes continuously decreasing in size in the ocelot gecko (Main et al., 2012; Koubová et al., 2014) and 2n = 50 in the painted turtle (13 pairs of macro chromosomes and 12 pairs of micros) (Badenhorst et al., 2015) (Figure 1B).

**FIGURE 3:**
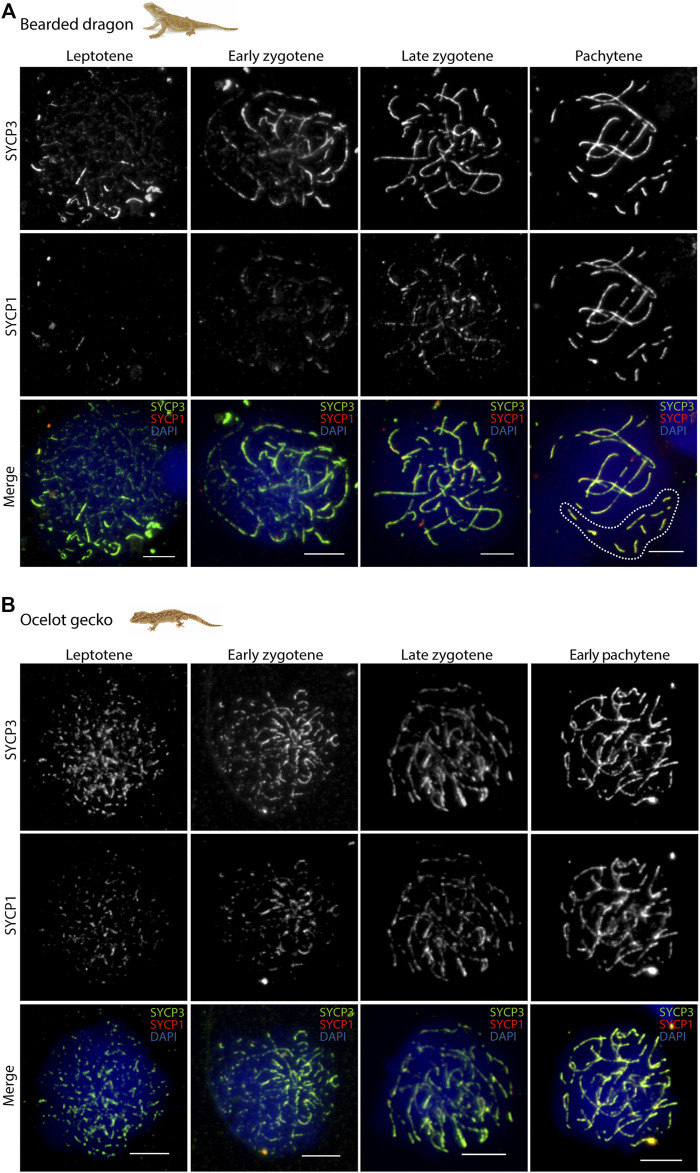
Synapsis dynamics during prophase I. Spermatocyte spreads labelled with antibodies against SYCP3 (green) and SYCP1 (red), counterstaining the DNA with DAPI (blue) for **(A)** the bearded dragon and **(B)** the ocelot gecko. Scale bar: 10 μm. White dashed circle: cluster of micro-chromosomes.

Axial elements of the synaptonemal complex labelled with anti-SYCP3 were used to classify spermatocytes into the different prophase I stages (leptotene, early zygotene, late zygotene and pachytene; Figure 3 and Supplementary Figure S1) as previously described (Alavattam et al., 2018). The proportion of thick (i.e., synapsis) and thin (i.e., unsynapsis) SYCP3 filaments were used to distinguish between earlier and later stages of zygotene spermatocytes. Zygotene spermatocytes with ≤ 50% of thick SYCP3 filaments (i.e., synapsis) were classified as “early stageˮ, whereas zygotene spermatocytes with > 50% of synapsis, were classified as “late stageˮ (Alavattam et al., 2018).

**FIGURE 4:**
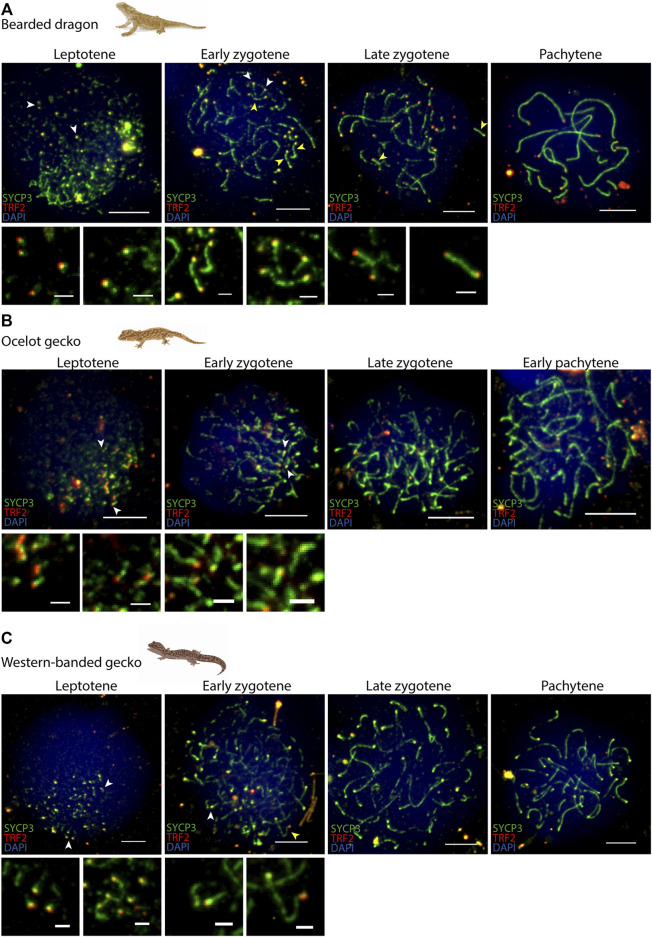
Telomere dynamics during prophase I. Spermatocyte spreads labelled with antibodies against SYCP3 (green) and TRF2 (red), counterstaining the DNA with DAPI (blue) for **(A)** the bearded dragon, **(B)** the ocelot gecko and **(C)** the western banded gecko. Scale bar: 10 μm and 2 μm (insets). White arrowheads: telomeres from which SC is beginning to assemble. Yellow arrowheads: completely associated micro-chromosomes (i.e., lateral elements of the SC completely assembled between both telomeric ends).

In all four species we observed short filaments of SYCP3 at leptotene, which represented the forming axial elements (Figure 3; Supplementary Figure S1). The general trend in all four species was for the chromosomes to start pairing at one pole of the cell at leptotene, forming the *bouquet*, from which SYCP3-positive filaments assembled from telomeres (Figure 4; Supplementary Figure S2). As prophase I progressed, axial elements become larger at zygotene, when synapsis between homologous chromosomes takes place, as revealed by SYCP3 and SYCP1 labelling (Figure 3; Supplementary Figure S1) (Alavattam et al., 2018). At pachytene, autosomes had completed synapsis. Remarkably, we found that micro-chromosomes completed synapsis earlier than macro-chromosomes, forming a discrete cluster (Figure 3A). This previously undescribed pattern was also highlighted by TRF2 immunostaining of telomeres, which revealed that some micro-chromosomes were fully synapsed in early zygotene (Figure 4; Supplementary Figure S2).

Moreover, phosphorylated RNA polymerase II (the active form of RNA pol II) was detected in spermatocytes of all four species, with increasing signal intensity through prophase I (Supplementary Figure S3), mirroring therian mammals (Page et al., 2012; Marín-Gual et al., 2022) and insects (Viera et al., 2017). The absence of transcriptional repression of any specific pair of chromosomes during pachytene (no antibodies against γH2AX yielded any positive staining) suggested that meiotic sex chromosome inactivation (MSCI) was absent in the four reptilian species, contrasting male mammals (Turner et al., 2005; Ruiz-Herrera and Waters, 2022). The absence of MSCI is not surprising because our reptile models either do not have sex chromosomes (the painted turtle, Valenzuela et al., 2014), or because males are the homogametic sex (the bearded dragon, Ezaz et al., 2005), or because sex chromosomes are likely poorly differentiated (the ocelot gecko and the western banded gecko, Keating et al., 2022; Rovatsos et al., 2019).

### DSBs dynamics in reptiles

**FIGURE 5:**
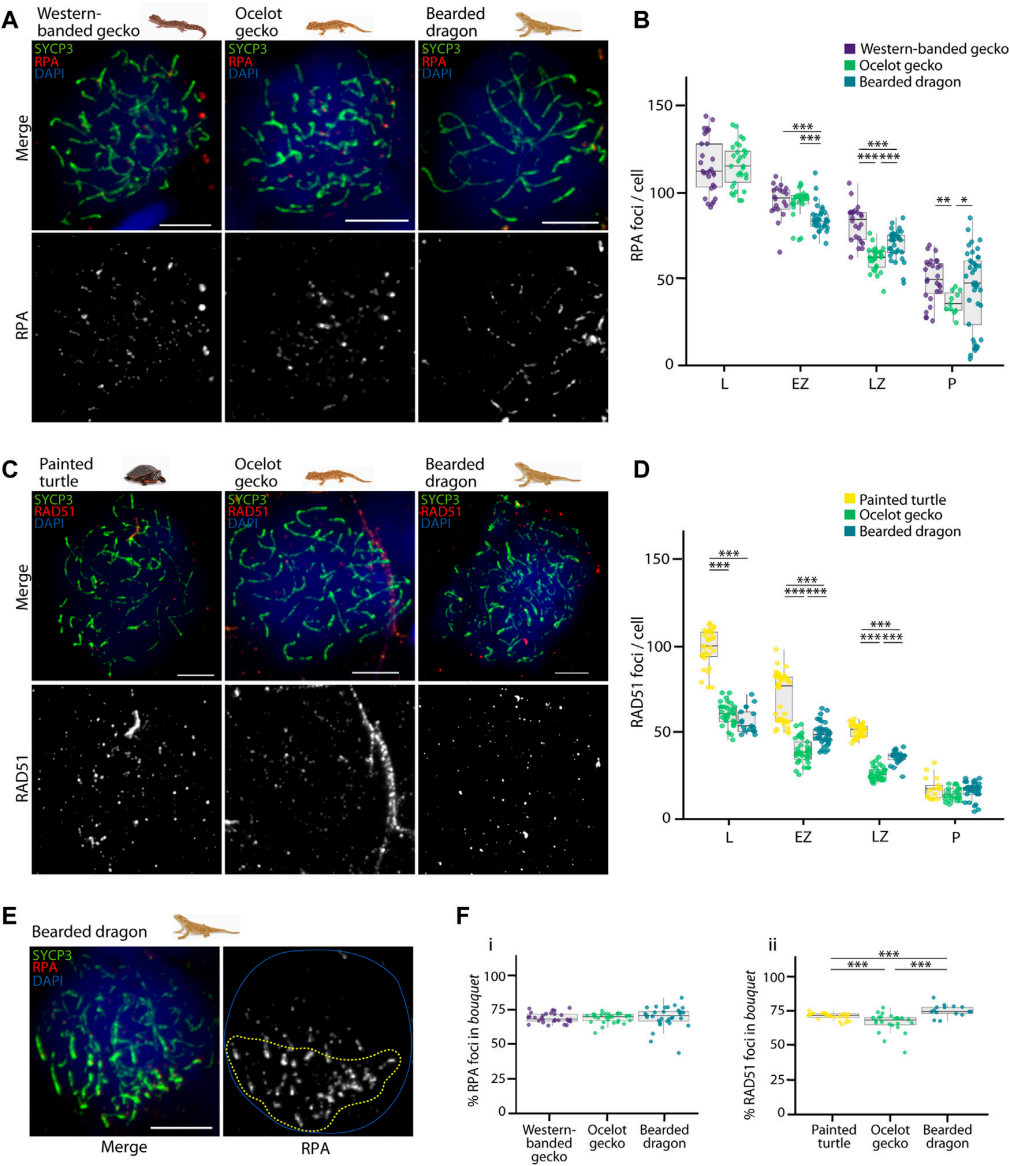
Double strand break formation dynamics during reptilian prophase I. **(A)** Late zygotene spermatocyte spreads labelled with antibodies against SYCP3 (green) and RPA (red), counterstaining the DNA with DAPI (blue) for the western banded gecko, the ocelot gecko and the bearded dragon. Scale bar: 10 μm. **(B)** Plot representing the number of RPA foci per cell detected at leptotene, early zygotene, late zygotene and pachytene. **(C)** Late zygotene spermatocyte spreads labelled with antibodies against SYCP3 (green) and RAD51 (red), counterstaining the DNA with DAPI (blue) for the painted turtle, the ocelot gecko and the bearded dragon. Scale bar: 10 μm. **(D)** Plot representing the number of RAD51 foci per cell detected at leptotene, early zygotene, late zygotene and pachytene. **(E)** Early zygotene spermatocyte spread labelled with SYCP3 (green) and RPA (red), counterstaining the DNA with DAPI (blue) in the bearded dragon. Dashed yellow circle: RPA foci detected in the *bouquet*. Blue circle: nuclei perimeter. **(F)** Plots representing the percentage of (i) RPA and (ii) RAD51 foci detected in the *bouquet* for the western banded gecko, the painted turtle, the ocelot gecko and the bearded dragon. Only cells with a well-defined *bouquet* were included in the analysis. The number of cells examined per species per stage, illustrated in panels **(B,D,F)**, are listed in Table 1. Mann-Whitney test (*p < 0.05, **p < 0.01 and ***p < 0.001). Legend: L, leptotene; EZ, early zygotene; LZ, late zygotene; P, pachytene.

We then analyzed the dynamics of DSB formation by immunodetection of the recombination proteins RPA (Replication Protein A) and RAD51 (Radiation Sensitive 51) (Figure 5; Supplementary Figures S4, S5) as no antibodies against MLH1 yielded any positive staining in reptile spermatocytes (data non shown). RPA binds to the 3’ DNA strand following DSBs formation, and is subsequently replaced by RAD51 and/or DMC1 by early prophase I (He et al., 1995; Keeney et al., 1997). As such, the number of RPA and RAD51 sites in early prophase is a proxy for the number of DSBs, as previously described for various mammalian taxa (Segura et al., 2013; Ruiz-Herrera et al., 2017; Marín-Gual et al., 2022).

We successfully detected RPA foci in spermatocyte spreads of the western banded gecko, the ocelot gecko, and the bearded dragon (Figures 5A,B; Supplementary Figure S4; Table 1). Both geckos had equivalent numbers of RPA foci at leptotene and early zygotene (Mann-Whitney test, *p* ≥ 0.05, Table 1). However, they had different RPA replacement dynamics, because the mean number of RPA foci was higher in the western banded gecko compared to the ocelot gecko by late zygotene (Mann-Whitney test, *p* < 0.001) and pachytene (Mann-Whitney test, *p* < 0.01) (Table 1). Furthermore, the RPA loading and replacement dynamics in the bearded dragon differed from both geckos, with lower RPA foci per cell at early zygotene (Mann-Whitney test, *p* < 0.001), intermediate values at late zygotene (Mann-Whitney test, *p* < 0.001) and higher RPA foci at pachytene compared to the ocelot gecko (Mann-Whitney test, *p* < 0.05) (Figure 5B; Table 1).

**TABLE 1:** Dynamics of DSB formation during prophase I in the four reptiles included in the study. Values indicate the average number of RPA or RAD51 foci per cell immunodetected in leptotene, early zygotene, late zygotene, and pachytene, as well as the fraction of the total foci per cell detected in the *bouquet*. Values in parenthesis indicate the number of cells analyzed in each case.

		Leptotene	Early zygotene	Late zygotene	Pachytene	% foci in *bouquet*
Mean RPA foci/cell	Western-banded gecko	115 (29)	95 (20)	82 (24)	49 (17)	69 (28)
	Ocelot gecko	115 (30)	94 (25)	61 (27)	36 (11)	69 (29)
	Bearded dragon	–	85 (32)	70 (32)	44 (41)	70 (32)
Mean RAD51 foci/cell	Painted turtle	103 (29)	75 (37)	56 (37)	22 (17)	70 (26)
	Ocelot gecko	65 (31)	44 (37)	31 (35)	19 (25)	66 (24)
	Bearded dragon	61 (17)	53 (36)	40 (24)	21 (34)	75 (15)

Similar dynamics (i.e., a decreasing numbers of foci as prophase I progressed) were detected for RAD51, which we detected in spermatocyte spreads of the painted turtle, the ocelot gecko and the bearded dragon (Figures 5C,D; Supplementary Figure S5; Table 1). Both lizards had equivalent values of RAD51 foci per cell at leptotene (Mann-Whitney test, *p* ≥ 0.05), whereas the mean was higher in the bearded dragon compared to the ocelot gecko by early zygotene (Mann-Whitney test, *p* < 0.001) and late zygotene (*p* < 0.001) (Table 1). In contrast, the painted turtle showed higher mean values of RAD51 foci per cell compared to both lizards at all stages of prophase I (Man-Whitney test, *p* < 0.001) except in pachytene (Mann-Whitney test, *p* ≥ 0.05) (Figure 5D; Table 1).

Remarkably, RPA and RAD51 loading followed similar dynamics, with both proteins accumulated in the *bouquet* region at early stages of prophase I (Figure 5E). Despite some variation among reptile species, between 66% and 75% of the total RPA and RAD51 foci per cell were detected in the *bouquet* (Figure 5F), indicating that DSB formation initiates at telomeres.

## Discussion

Our work represents a comparative study of the dynamics of the spermatogenic cycle and prophase I progression in reptiles, with an emphasis on chromosome pairing and the formation and repair of meiotic DSBs.

### Continuous spermatogenic cycle in the bearded dragon and the painted turtle

Spermatogenesis in vertebrates follows two main arrangements in the seminiferous epithelia: (i) cystic, in which developing germ cell syncytia are individually encapsulated by Sertoli cells as observed in fish and amphibians, and (ii) noncystic, where spermatogenesis takes place in seminiferous tubules (reptiles, birds, and mammals) (Schulz et al., 2010; Sousa et al., 2014). In species with noncystic spermatogenesis, the seminiferous epithelium is the building block of seminiferous tubules, which are primarily composed of Sertoli cells and germ cells. Then, germ cell differentiation takes place in a continuous manner with a species-specific time interval (e.g., 8.6 days in mice and 16 days in humans, Russell et al., 1993).

Both the histological and cytological characterization presented here for reptiles revealed that spermatogenesis progression is noncystic and highly conserved with respect to cell morphology and distribution. Analysis of the histological distribution of different germ cell types within the seminiferous epithelia revealed that both the bearded dragon and the painted turtle showed similar patters to those described in eutherian mammals (Russell et al., 1993) and other reptiles (Gribbins, 2011; Sousa et al., 2014). In fact, all amniotes described to date present similar testis structure and organization, although differences have been reported in terms of reproductive strategy and behaviour, including both continuous and seasonal breeding (Gribbins et al., 2003).

In temperate and subtropical lizards, the testicular cycle is divided in two phases: (i) the regenerative phase, which occurs in the spring, and (ii) the degenerative phase, that begins in summer (Mayhew and Wright, 1970; Amey and Whittier, 2000a). So, there is a cycle of hypertrophy and regression of reproductive organs. Previous studies in the eastern bearded dragon *Pogona barbata* classified testis as (i) regressed (only spermatogonia present), (ii) developing (spermatocytes or spermatids present), and (iii) spermiogenic (spermatogenesis and mature sperm present) (Amey and Whittier, 2000a). Consistently, the observations made here for the central bearded dragon agree with the observation of maximum spermatogenic activity in spring, followed by the cessation of spermatogenesis directly after the breeding period and testicular recrudescence in February (late summer). Both bearded dragons and the painted turtle are seasonal breeders (Gibbons, 1968; Amey and Whittier, 2000b) and since samples were collected at the end of the mating season for both species, our results showed that germ cells enter the spermatogenic cell cycle continually during the reproductive season, as all cell stages were found in the seminiferous epithelium.

### Meiosis progression is highly conserved in reptiles

We found that the progression of prophase I was highly conserved among the reptiles examined. In most species, chromosomes were organized into chromosome axes but were not yet synapsed at leptotene. This was coupled with the formation of DSBs (Zickler and Kleckner, 2015). In our target reptiles, chromosome axes were detected as short stretches of SYCP3 and SYCP1 signal adjacent to the telomes, mirroring the patterns previously described in zebrafish (Blokhina et al., 2019; Imai et al., 2021). Moreover, telomeres clustered to one side of the nucleus forming the *bouquet*, presumably promoting homologous pairing and synapsis. The *bouquet* first appeared from leptotene to late zygotene, depending on the species. This feature is shared with zebrafish, suggesting that early telomere clustering is ancestral and has been retained over almost 400 million years of vertebrate evolution.

Interestingly, micro-chromosomes completed synapsis earlier than macro-chromosomes, forming discrete clusters concurrent with the *bouquet*. This suggests that the *bouquet* facilitates SYCP3 loading and synapsis of homologous chromosomes (both macro and micro-chromosomes) from the telomeres towards the central regions of the chromosomes, at the same time as micro-chromosome synapsis. Complete homologous synapsis of micro-chromosomes was observed from early zygotene to pachytene. This was coupled with a polarization of DSBs towards telomeres, a notable difference compared to some mammal species where DSBs are distributed more homogenously across the genome as the SC is being assembled (Ruiz-Herrera et al., 2017; Marín-Gual et al., 2022), although DSBs and COs have been reported to be enriched at sub-telomeric regions in human males (Barlow and Hultén, 1998; Khil and Camerini-Otero, 2010; Pratto et al., 2014).

The clustering of telomeres during early prophase I extends previous cytological and genomic studies where micro-chromosomes in reptiles tend to clump centrally in mitotic and meiotic metaphase I (Waters et al., 2021), resulting in higher inter-chromosomal genomic interactions between micro-chromosomes than for macro-chromosomes (Waters et al., 2021). This results in micro-chromosomes forming a structural and functional domain that is maintained in germ cells, probably facilitating homology search and DSBs formation. As most sauropsids are characterized by very conservative genomes (with few macro-chromosomes and up to many micro-chromosomes) (Valenzuela and Adams, 2011; Waters et al., 2021), we hypothesize that meiotic patterns described herein will apply widely in this clade.

### Low meiotic DSB rates in reptiles

Remarkably, reptiles showed lower levels of DSBs than eutherian mammals. Although variable among species, between 200 and 300 DSBs per cell (mean values) occur genome-wide during leptotene in eutherian mammals (Segura et al., 2013). Marsupials show the lowest recombination rates in mammals with less than 150 RPA foci per cell in zygotene (Zenger et al., 2002; Samollow et al., 2004; Marín-Gual et al., 2022). This contrasts with our results where fewer RPA foci (from 95 to 85 per cell) and RAD51 foci (from 75 to 44 per cell) (a proxy for DSBs) were observed in early zygotene in reptiles. A closer inspection of the data revealed that squamates (geckos and the bearded dragon) showed equivalent values of DSBs in early stages of prophase I, and lower than turtles, the sister taxon to archosaurs (birds plus crocodilians). Two biological, non-mutually exclusive alternatives could explain these observations: differences in DSBs per cell observed between squamates and turtles are due to (i) contrasting chromosome number and size between the taxa examined, or (ii) these lineages differ in the genetic determinants of DSBs induction.

The first alternative agrees with previous cytological data. Specifically, studies in disparate taxa show that the total number and distribution of COs (and also initial meiotic DSBs) on a specific chromosome depends on several factors, such as chromosome size, and an individual’s sex and age (Pardo-Manuel De Villena and Sapienza, 2001; Lynn et al., 2004; Garcia-Cruz et al., 2011; Ruiz-Herrera et al., 2017; Wang et al., 2019). Larger chromosomes tend to accumulate more COs (but see recent data in yeast, Murakami et al., 2021), and each chromosome arm generally presents at least one CO (the obligatory chiasmata) (Sun et al., 2017). Thus, because both geckos and the bearded dragon have lower diploid numbers (2n = 32–36) than the painted turtle (2n = 50), differences in meiotic DSBs (COs were not reported in this study) are expected among groups. This rationale would also apply to birds, which possess high chromosome numbers (typically 2n = 80, Ruiz-Herrera et al., 2012; Waters et al., 2021). In birds, cytological analyses of COs (meiotic DSBs are unreported) are restricted to species from the domesticated groups Galloanserae and Passeriformes, in which recombination rates are higher (from 1.8 cM/Mb to 2.6 cM/Mb; del Priore and Pigozzi, 2020) than those reported for mammals (from 0.18 cM/Mb to 1.78 cM/Mb; Segura et al., 2013). Moreover, birds show little variation in recombination rates between groups (del Priore and Pigozzi, 2020), and thus are not lineage-dependent (unlike mammals, Segura et al., 2013), perhaps related to high genome conservation (Waters et al., 2021).

The second hypothesis derives from the observation that recombination rates may vary by the presence of different genetic determinants of DSBs induction, such as PRDM9 (Mihola et al., 2009; Myers et al., 2010; Grey et al., 2011; Vara et al., 2019). PRDM9 is a meiotic-specific histone (H3) methyltransferase with a C-terminal tandem repeat zinc finger (ZnF) domain that adds H3K4me3 marks at nucleosomes close to DSBs in early meiosis. This process genetically determines recombination hotspots (Mihola et al., 2009; Baudat et al., 2010; Grey et al., 2011). Experimental work has shown that PRDM9 provokes changes in local chromatin structure that tend to position nucleosomes in ways that increased overall accessibility (Yamada et al., 2020). Moreover, in the absence of PRDM9, DSBs tend to form in gene promoter regions (Brick et al., 2012; Baker et al., 2015; Lange et al., 2016). PRDM9 is present in most mammals (with the exception of canids, Muñoz-Fuentes et al., 2011), but substantial allelic variability was described in natural populations, especially in rodents (Buard et al., 2014; Capilla et al., 2014; Vara et al., 2019) contributing to speciation (Smagulova et al., 2016). Most importantly, PRDM9 gene was lost at least 13 times independently in vertebrates, including in birds, some snakes, and lizards (Cavassim et al., 2022). Although bearded dragons have a complete PRDM9 (Cavassim et al., 2022), little is known regarding geckos and turtles. It is tempting to speculate the existence of yet to be discovered genetic determinants of recombination across vertebrates, and that one (or more) of these might be responsible for reduced recombination rates observed in the species herein. Further research is needed to fully test these hypotheses.

### Limitations of the study

As the use of non-model species can be challenging, future studies with a larger number of animals per species will be desirable to capture inter-individual variability in recombination rates. Additionally, here we report results obtained with two early markers of meiotic DSBs (RAD51 and RPA) in four different reptile species, but the use of direct measures of COs were precluded. Thus, future research focussed on MLH1 foci (a marker of COs) together with the analysis of chiasmata in metaphase I will provide a comprehensive view of the recombination dynamics in reptiles.

## Conclusion

Overall, our findings provide new insights into meiotic chromosome dynamics and double strand break formation during reptile spermatogenesis. Shared histological patterns observed between squamates and turtles suggest that they represent the ancestral state. However, future research across more species is warranted to asses conservation of this ancestral pattern across other sauropsids. Understanding the intricacies of the mechanisms regulating chromosome synapsis, recombination and segregation during meiosis progression across vertebrates will further determine the genomic basis of biodiversity, and how it may be affected by ecotoxicological and other environmental changes.

## Data Availability

The original contributions presented in the study are included in the article/Supplementary Material, further inquiries can be directed to the corresponding author.

## Supplementary material

Supplementary Figure 1: Synapsis dynamics during prophase I. Spermatocyte spreads labelled with antibodies against SYCP3 (green) and SYCP1 (red), counterstaining the DNA with DAPI (blue) for **(A)** the western-banded gecko and **(B)** the painted turtle. Scale bar: 10 μm.

Supplementary Figure 2: Telomere dynamics during prophase I. Spermatocyte spreads labelled with antibodies against SYCP3 (green), SYCP1 (red) and TRF2 (blue) for the bearded dragon. Scale bar: 10 μm and 2 μm (insets). White arrowheads: telomeres from which SC is beginning to assemble. Yellow arrowheads: completely synapsed micro-chromosomes (i.e., lateral and central elements of the SC completely assembled between both telomeric ends). Insets (bottom row panels) show micro-chromosomes completely assembled.

Supplementary Figure 3: Transcriptional dynamics during prophase I. Spermatocyte spreads labelled with antibodies against SYCP3 (green) and RNApol II (red) for **(A)** the bearded dragon, **(B)** the ocelot gecko, **(C)** the western banded gecko and **(D)** the painted turtle. Scale bar: 10 μm.

Supplementary Figure 4: RPA dynamics during reptilian prophase I. Spermatocyte spreads labelled with antibodies against SYCP3 (green) and RPA (red), counterstaining the DNA with DAPI (blue) for **(A)** the western banded gecko, **(B)** the ocelot gecko and **(C)** the bearded dragon. Scale bar: 10 μm.

Supplementary Figure 5: RAD51 dynamics during reptilian prophase I. Spermatocyte spreads labelled with antibodies against SYCP3 (green) and RAD51 (red), counterstaining the DNA with DAPI (blue) for **(A)** the painted turtle, **(B)** the ocelot gecko and **(C)** the bearded dragon. Scale bar: 10 μm.
