# Supplementary figures and images for: Meiotic chromosome dynamics and double strand break formation in reptiles
Source: Front Cell Dev Biol. 2022 Oct 12;10:1009776. doi: 10.3389/fcell.2022.1009776 (PMC9597255; doi:10.3389/fcell.2022.1009776)

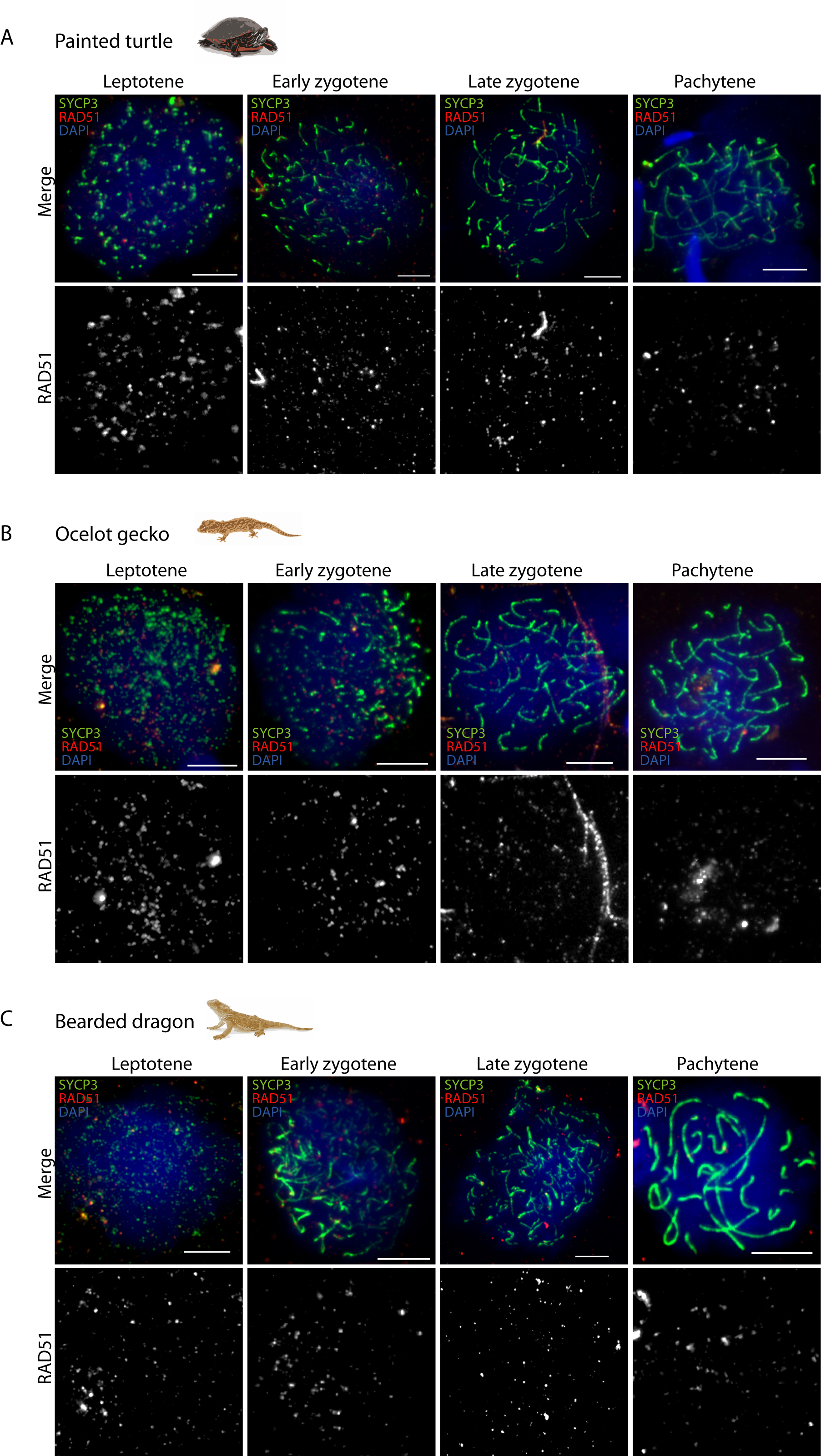

Supplement: Supplementary file 1 [file Image5.jpg]

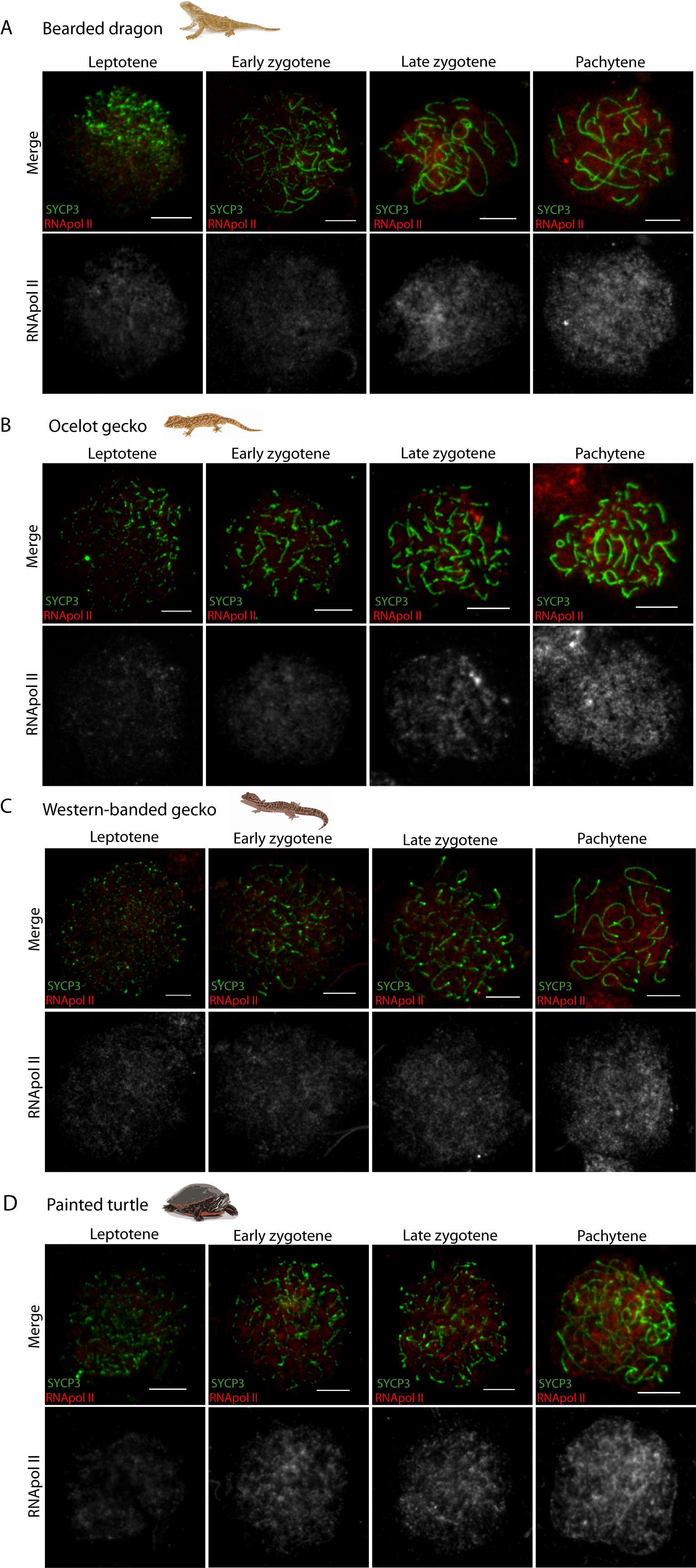

Supplement: Supplementary file 2 [file Image3.jpg]

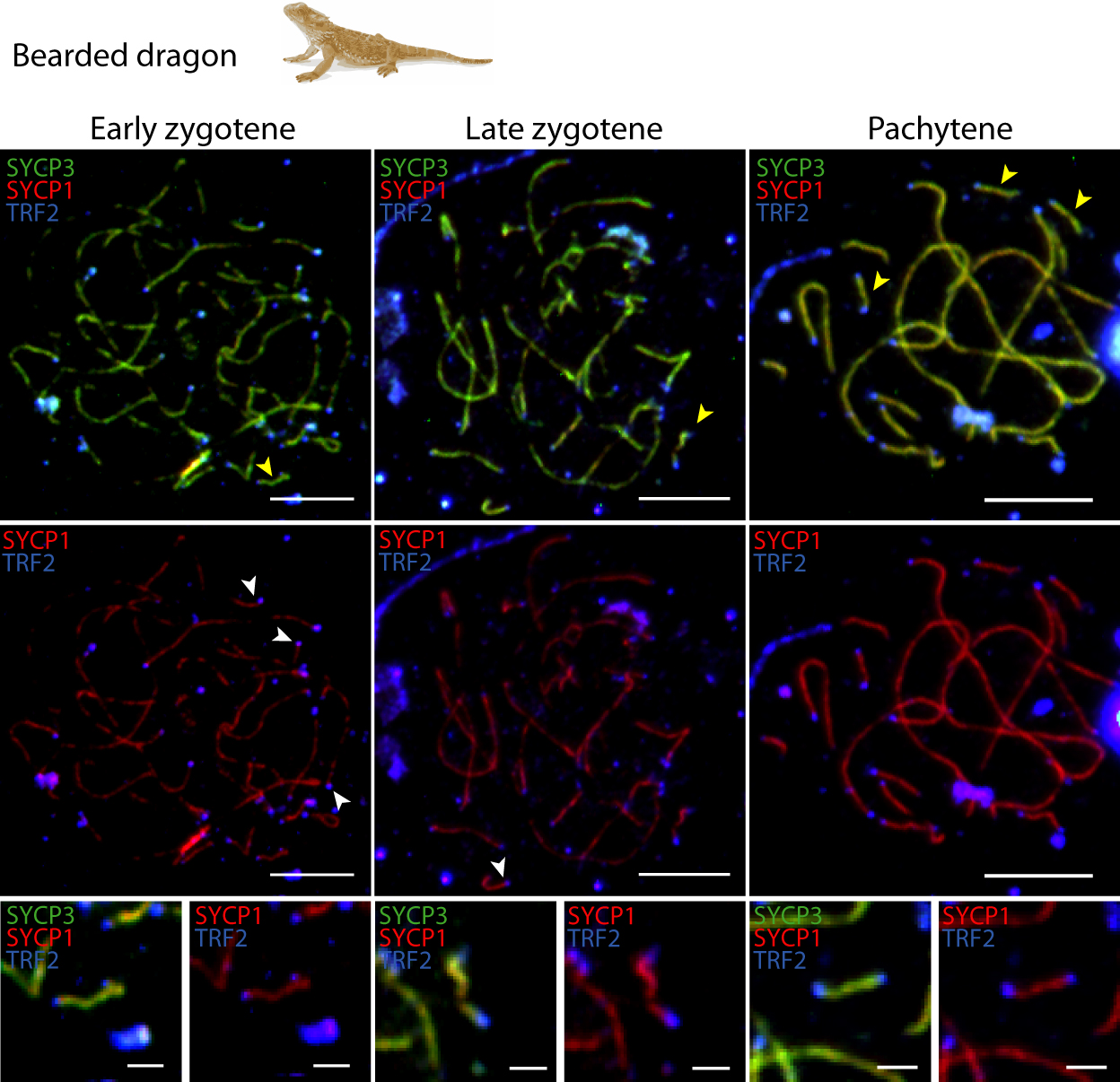

Supplement: Supplementary file 3 [file Image2.jpg]

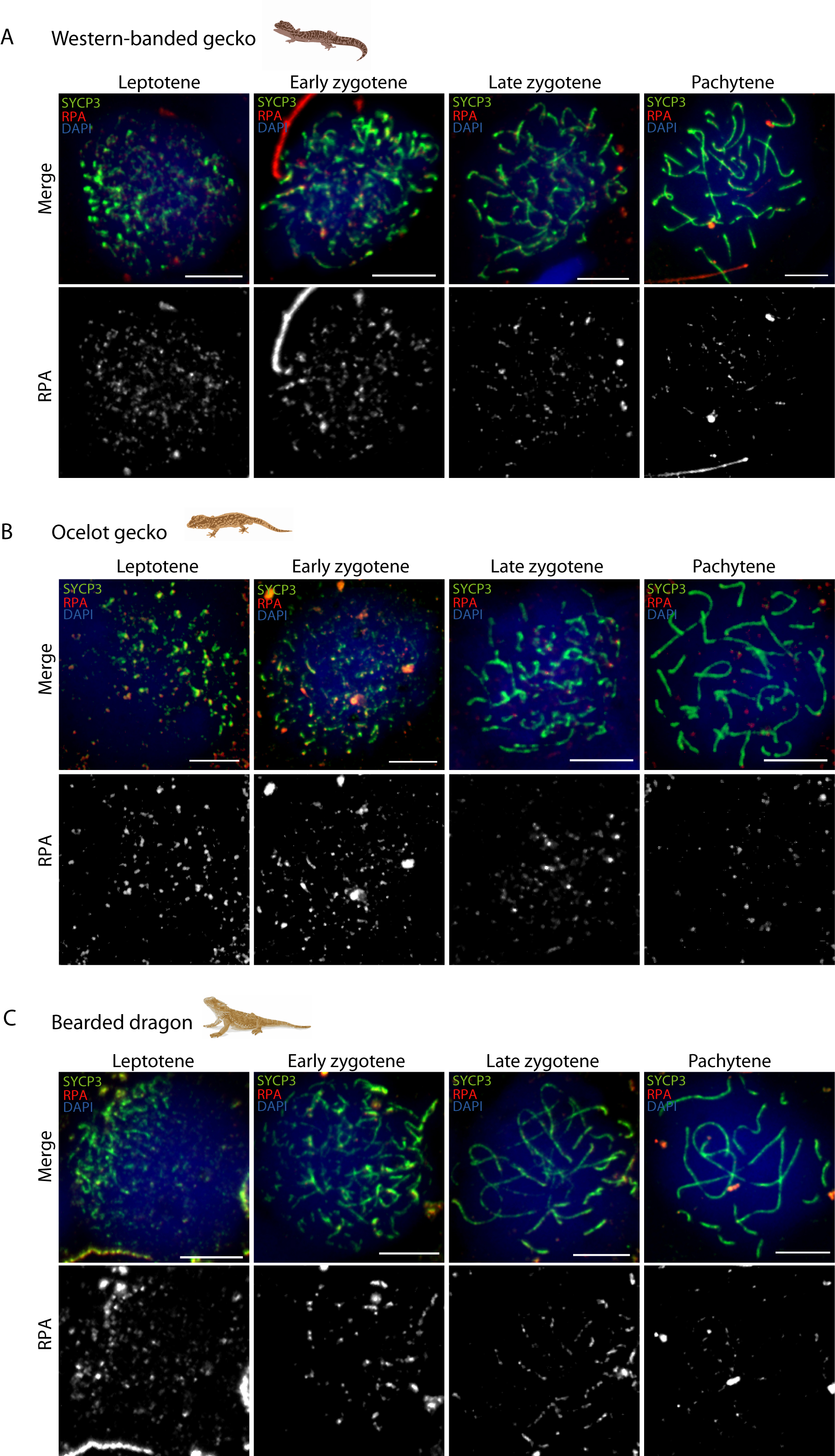

Supplement: Supplementary file 4 [file Image4.jpg]

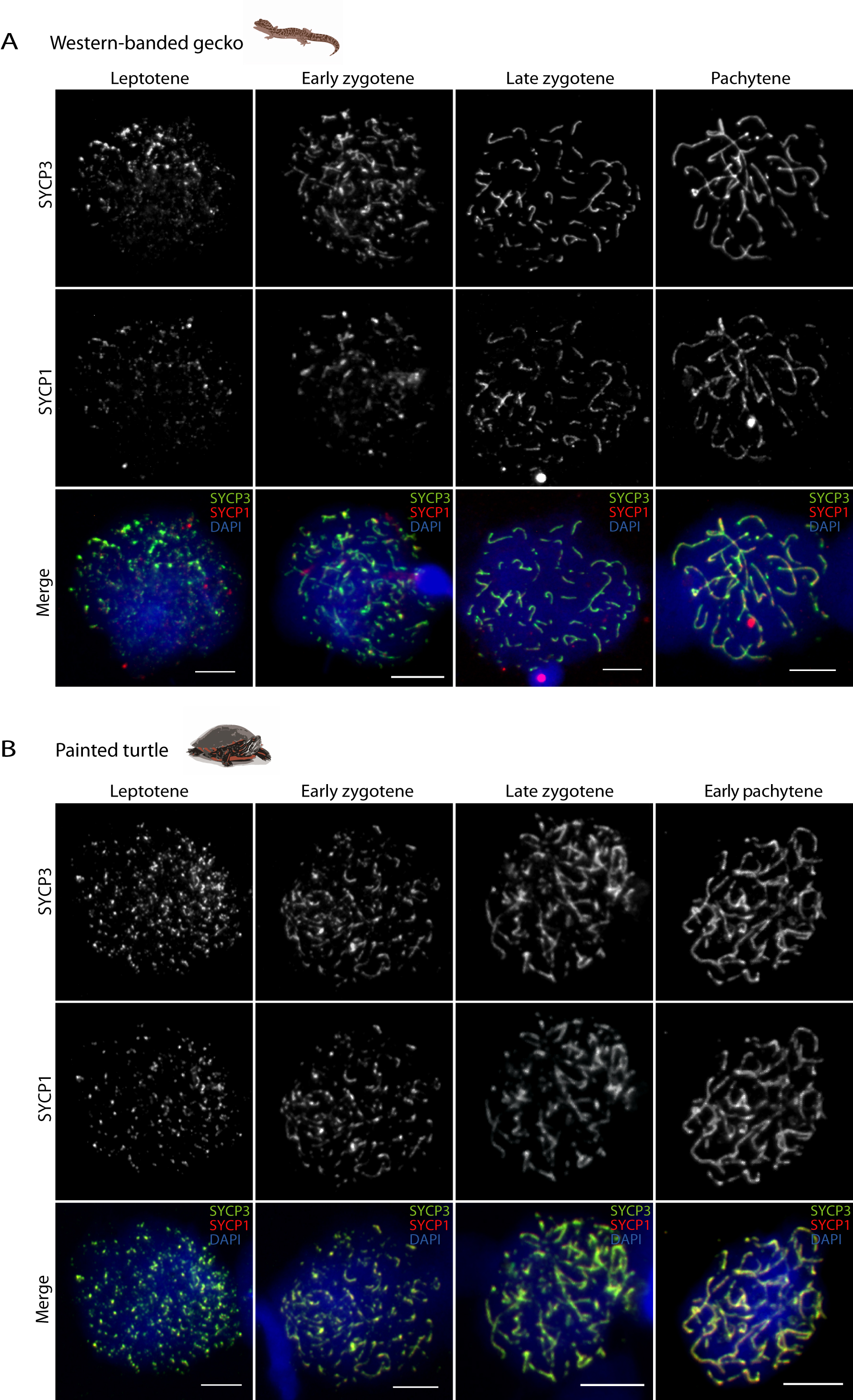

Supplement: Supplementary file 5 [file Image1.jpg]
